# Infectiousness of pigs infected by the Porcine Reproductive and Respiratory Syndrome virus (PRRSV) is time-dependent

**DOI:** 10.1186/1297-9716-43-69

**Published:** 2012-10-12

**Authors:** Céline Charpin, Sophie Mahé, André Keranflec’h, Catherine Belloc, Roland Cariolet, Marie-Frédérique Le Potier, Nicolas Rose

**Affiliations:** 1 Anses – laboratoire de Ploufragan-Plouzané, BP53, 22440, Ploufragan, France; 2 UMR BioEpAR, Oniris, INRA, LUNAM, BP 40706, 44307, Nantes Cedex 03, France

## Abstract

The time-dependent transmission rate of Porcine Reproductive and Respiratory Syndrome Virus (PRRSV) and the correlation between infectiousness, virological parameters and antibody responses of the infected pigs were studied in experimental conditions. Seven successive transmission trials involving a total of 77 specific pathogen-free piglets were carried out from 7 to 63 days post-inoculation (dpi). A semi-quantitative real time RT-PCR was developed to assess the evolution of the viral genome load in blood and nasal swabs from inoculated and contact pigs, with time. Virus genome in blood was detectable in inoculated pigs from 7 to 77 dpi, whereas viral genome shedding was detectable from nasal swabs from 2 to 48 dpi. The infectiousness of inoculated pigs, assessed from the frequency of occurrence of infected pigs in susceptible groups in each contact trial, increased from 7 to 14 dpi and then decreased slowly until 42 dpi (3, 7, 2, 1 and 0 pigs infected at 7, 14, 21, 28 and 42 dpi, respectively). These data were used to model the time-dependent infectiousness by a lognormal-like function with a latency period of 1 day and led to an estimated basic reproduction ratio, *R*_0_ of 2.6 [1.8, 3.3]. The evolution of infectiousness was mainly correlated with the time-course of viral genome load in the blood whereas the decrease of infectiousness was strongly related to the increase in total antibodies.

## Introduction

Porcine Reproductive and Respiratory Syndrome (PRRS) is responsible for reproductive disorders in sows and respiratory problems in piglets and growing pigs [1,2]. This disease leads to considerable economic losses as well as the extensive use of antibiotics to control bacterial secondary infections in pig production. In 2011, the estimated annual economic losses caused by PRRS in the American swine industry were approximately 664 million dollars [3]. In France, the situation regarding spatial distribution of the disease is heterogeneous. Some areas are almost disease-free (southern Normandy, Pays de la Loire [4]) whereas the disease is highly prevalent in densely pig populated areas like Brittany where more than 50% of French pigs are raised [5,6].

PRRS is caused by a small, enveloped positive-stranded RNA virus, PRRSV, which belongs to the family *Arteriviridae*, genus Arterivirus [7]. Two main genotypes with only 60–70% nucleotide homology, the so-called North American and European PRRSV genotypes, are clearly distinguished [8-12]. There are also regional differences within each genotype although a relative homogeneity has been reported within countries [11]. The special feature of PRRSV infection as compared to other viral infections is the prolonged viremia and subsequent virus shedding [13]. The infectious agent can survive for a long time in an infected pig, and animals remain contagious even when they recover from clinical disease. The estimated infectious period, according to serological prevalence data in a recently infected farrow-to-finish farm, was 56 days [14]. Prolonged duration of virus shedding has also been described (detection from oropharyngeal samples up to 156 days post-infection (dpi) [15]. Using a modeling approach with an estimated 56 day-long infectiousness, Nodelijk et al. [14] found that it took about 6 years to eliminate the infection from a herd of 115 sows without any virus reintroduction and about 80 years if the farm size was twice as large. In another more recent study [16], based on the same assumed infectious period (56 days), the duration of persistence seemed to be influenced by the size of the herd, but also by the separation of gilts and sows.

However, little is known about the horizontal transmission of PRRSV in growing pigs. In the previous models, transmission since infection was assumed to be constant with time. In the specific case of this viral infection with prolonged viral shedding, this assumption might not hold. After experimental infection, the genome load in the blood is detectable about 3 days post-inoculation and then increases rapidly until day 14, after which it decreases gradually and may persist for several weeks. However, the detection of PRRSV genome load in sera or nasal swabs cannot be considered as evidence of infective viral particles. The only evidence of infectiousness in a typical infected pig is its ability to infect a susceptible one after a period of contact.

The aim of the present work was therefore to assess the dynamics of infectiousness in inoculated pigs in terms of time elapsed since inoculation, under experimental conditions and then to correlate this infectiousness with virological parameters and antibody responses of the infected pigs.

## Materials and methods

### Animals and experimental design

Seventy-seven specific pathogen-free (SPF) piglets free from PRRS virus (and without any maternal antibody against this virus) and also from PCV-2, derived from the Anses (Agence nationale de sécurité sanitaire des aliments, de l’environnement et du travail) SPF herd were used. In this herd, piglets are not submitted to tail docking nor ear notching and they do not receive any medication except iron injection after birth. All the piglets were individually identified with an ear-tag.

**Figure 1:**
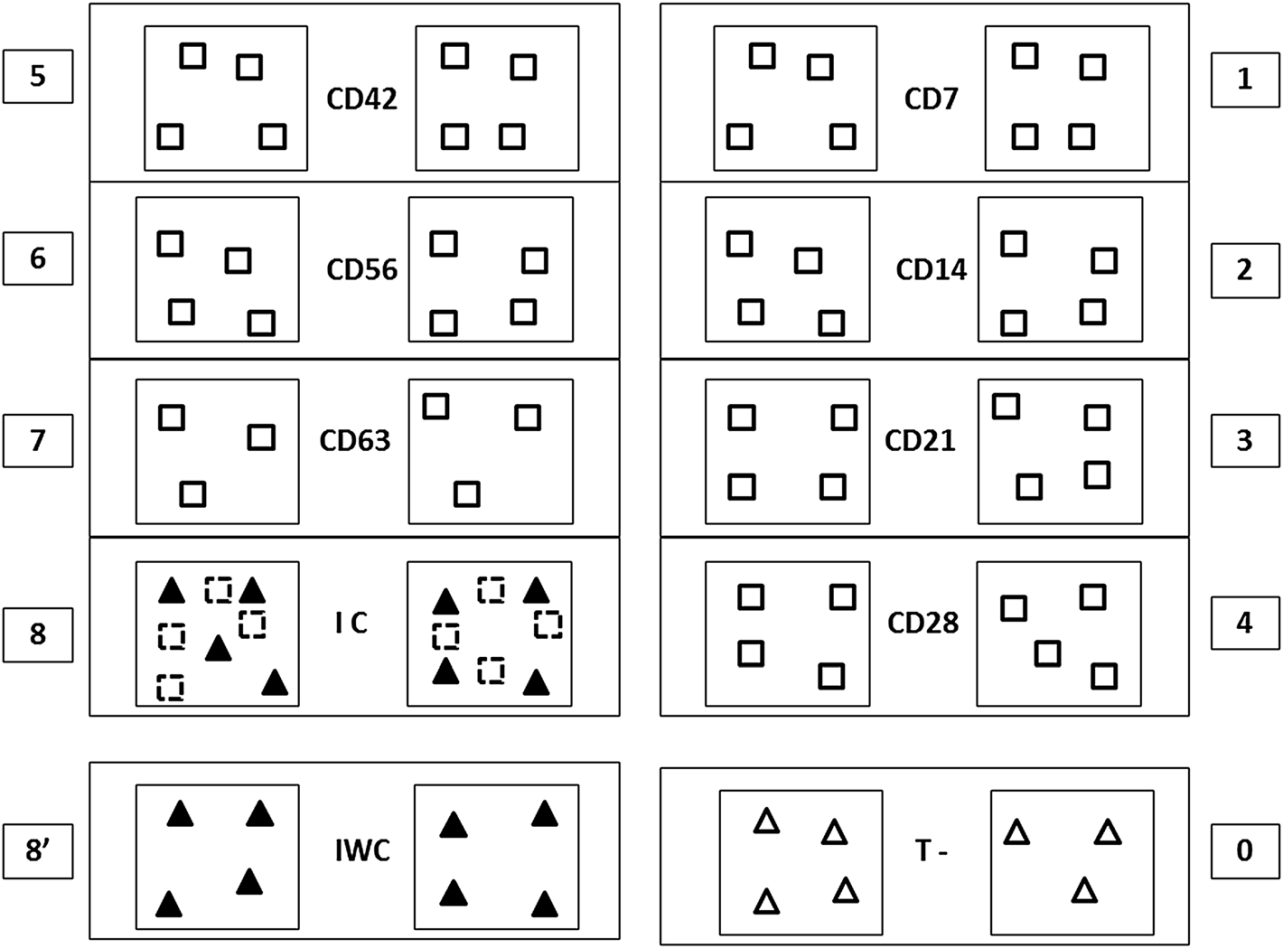
**Experimental design of thetransmission experiment.** Black triangles: infected piglets, white triangles: negative control piglets, white squares: contact piglets. CD7 to CD63: contact groups from 7 to 63 dpi. IC: Inoculated with contacts, IWC: inoculated without contact. T-: control group.

Seven pigs were kept as negative controls (inoculated with 5 mL of PBS) in room 0 and the 70 remaining pigs were randomly assigned to 9 other groups housed in 9 separate rooms in the air-filtered level-3 biosecurity facilities, each room containing two pens of 4 pigs (3 for the CD63 group) with a polyethylene separation between the pens (Figure 1).

Pigs in rooms 8 (designated “IC” for “Inoculated with Contact”) and 8’ (designated “IWC” for “Inoculated Without Contact”) were inoculated with PRRS virus at 5 weeks of age. IWC pigs were kept as infected controls. Pigs in room 8 (IC) were mingled successively with susceptible pigs from rooms 1 to 7. The seven groups of 8 SPF pigs (designated “CDX” for “Contact Day X”): CD7, CD14, CD21, CD28, CD42, CD56 and CD63 were mingled with the seeder group (IC) at 7, 14, 21, 28, 42, 56 and 63 dpi, respectively. Once mingled, they remained for 2 days with the seeders and were then returned to their original room to be monitored for PRRSV infection. Pigs were housed on flat decks (with a slatted floor 1 meter above the floor of the facility). The floor of the flat deck and of the facilities was cleaned thoroughly every day and just before receiving the contact pigs in order to preclude environmental contamination. The experiment was performed in accordance with EU and French regulations on animal welfare in experimentation. The protocol was approved by the Anses/ENVA/UPEC ethical committee (agreement #16 to the National committee for ethic in animal experimentation).

### Inoculum

Infected pigs were inoculated by intranasal route on day 0 with 5 mL of a PRRSV suspension (10^5^ TCID_50_ mL^-1^) derived from the Spanish strain 218 (Genotype I, 97% homology with the Lelystad reference strain) [17]. Viruses were grown in primary porcine alveolar macrophage cultures and used after limited passage.

### Observation and sampling

Pigs were examined daily for clinical signs (rectal temperature, coughing, sneezing, individual weight, feed consumption) until they were euthanized at the end of the experiment (98 dpi) in the case of inoculated seeder and control pigs (IC and IWC), or at least 28 days post-mingling in the case of contact pigs. Blood samples were taken weekly for PRRSV serology and genome detection. Nasal swabs were taken twice a week for PRRSV genome detection. Euthanasia was carried out by anesthesia (sodium thiopental injection (1g/50 kg)) followed by exsanguination. All pigs were necropsied and their organs examined.

### Total antibodies

The PRRSV antibodies in the weekly collected sera were detected using the ELISA kit HerdChek* PRRS X3 (IDEXX, Liebefeld-Bern, Switzerland) according to the manufacturer’s instructions. Samples with an S/P ratio above 0.4 were considered positive for PRRSV antibodies.

### Neutralizing antibodies

PRRSV-specific neutralizing antibodies were quantified by viral neutralization on MARC-145 cells against 100 DCP_50_ of the Spanish PRRSV strain 218 (previously adapted to MARC-145 cells). The tests were performed in duplicate on non-inactivated serum. Briefly, 50 μL of total serum were diluted in series of 1 to 1/2048 in MEM, then mixed with 50 μL of 100 DCP_50_/50 μL of virus and incubated for 1 h at 37°C (+/- 2°C), 5% CO_2,_ before adding 150 μL of MARC-145 cells (500 000 cells/mL). The plates were then incubated for 5 days at 37°C (+/- 2°C), 5% CO_2_. Cytopathic effects (CPE) were sought from the third day of incubation and the final reading of CPE was done after the fifth day of incubation. A control serum (addition of 50 μL of MEM instead of 50 μL of virus), a negative control serum (SPF pig serum) and a positive control serum (serum from a hyperimmunized pig) were included in each test. The serum titer was estimated by the Kärber method [18] and the neutralizing doses (ND_50_) were expressed as Log10-transformed values of neutralizing antibody titers.

### Semi-quantification of PRRSV genome load by real-time RT-PCR (rRT-PCR)

Viral RNA was extracted from EDTA-stabilized blood and nasal swabs using the commercial extraction kit Viral DNA/RNA Isolation-NucleoSpin® 8 Virus (MACHEREY-NAGEL, Düren, Germany) and Total RNA Isolation-NucleoSpin® 8 RNA (MACHEREY-NAGEL, Düren, Germany), respectively. Negative controls (with PBS instead of the sample) and a positive control (with the PRRSV strain instead of the sample) were included in each extraction.

A semi-quantitative rRT-PCR was developed to assess the evolution of the viral genome load in blood and nasal swabs from inoculated and contact pigs, with time. The ORF-7 sequence was chosen as target for virus detection being the most conserved region of the PRRSV genome between different genotypes. Beta-actin shows a good stability of expression in pig tissues [19]. It was therefore chosen as the internal control and normalizer gene for relative quantification of the genome viral load [20].

The PRRSV genome load was estimated by a real-time RT-PCR based on TaqMan technology. Briefly, the designed PRSSV- specific primers 5’- AACGYTCCCTCTGCTTGC-3’ and 5’- CTCAACCTGAAAACTGACCTTCC-3’ target the PRRSV ORF7 region and allow amplification of this fragment. The TaqMan probe 5’- CGATCCAGACGGCTTTYAATCAAGGCG-3’ was labeled with the fluorescent reporter dye FAM (6-carboxyfluorescein) at the 5’ end and with the nonfluorescent quencher (TAM) associated with the minor groove binder at the 3’ end.

Beta-actin was used as the internal control and normalizer gene for semi-quantification of the viral genome load. The designed Beta-actin–specific primers 5’-CTCGATCATGAAGTGCGACGT-3’ and 5‘-GTGATCTCCTTCTGCATCCTGTC-3’ target the beta-actin and allow amplification of this fragment. The TaqMan probe 5’- ATCAGGAAGGACCTCTACGCCAACACGG -3’ was labeled with the fluorescent reporter dye TET at the 5’ end and with the nonfluorescent quencher (BHQ1) associated with the minor groove binder at the 3’ end [20].

Real time RT-PCR was performed using SuperscriptTM III Platinum® One-Step Quantitative RT-PCR System (Carlsbad CA, USA) reagents. The 20 μL reaction mixture consisted of 1.5 μL of RNase free water, 12.5 μL of Taq SuperScript III RT-PCR master mix (MgSO4, and stabilizing dNTPs), 1 μL of enzyme, 2.5 μL of ORF7 mix FAM at 2 pmol/μL and 2.5 μL of beta-actin mix TET at 2 pmol/μL. The RT-PCR was performed on a Bio-Rad thermocycler PTC-0200 DNA Engine® Thermal Cycler (Bio-Rad) according to the following program: 50°C for 30 min, 94°C for 2 min followed by 45 cycles of 94°C for 15 s and 60°C for 30 s.

A negative control (with water instead of sample) and a positive control (with RNA extracted from the strain of PRRSV) were added to each run of rRT-PCR.

The specificity of the rRT-PCR was tested with other specific pathogens of pigs (swine influenza virus, Classical swine fever virus, African swine fever virus, BVD virus, Aujeszky disease virus and Mycoplasma). For each sample, the Ct (threshold cycle) obtained for the PRRS virus was normalized with the beta-actin Ct of the same sample, to obtain the normalized Ct of PRRS virus for a given sample. The relative amount of PRRSV RNA was then calculated for each sample, in relation to the sample corresponding to the smallest amount of virus genome in blood or nasal swabs, using the method of ΔΔCt and the equation R = (1 + E) ^Cti normalized-Ctj normalized^ = (1 + E) ^-∆∆Ct^[20-22], with E, the efficiency of the PCR, R the ratio between the amounts of PRRSV RNA in sample i and sample j containing the smallest amount of PRRSV RNA in the category of sample analyzed (blood or nasal swab). The results are expressed as log base 2.

### Estimation of the transmission parameter related to time since inoculation (β(τ))

The experiment relies on the fact that the transmission parameter (β) is not constant but varies with time since inoculation. Let τ and β(τ) be the time since inoculation and infectious potential of inoculated animals, respectively.

β(τ) is the mean number of pigs that could be infected by an inoculated pig at time τ after inoculation. Hence, the number of individuals potentially infected by a single infectious pig over a time interval of [t_0_, t_1_ is given by: $\int_{t0}^{t1}\beta \left(\tau \right)d\tau$. Thus, the basic reproduction ratio *R*_0_, which is equal to the average number of infected individuals produced by a single infectious one during its entire period of infectiousness, can be computed by integrating β(τ) over the entire infectious period, or equivalently over the period for which β(τ) is strictly positive. The probability of one pig escaping infection during a 2 day contact period (between t_i_ and t_i+1_) is given by $\text{qi}=\exp\left(-I\int_{\mathrm{ti}}^{ti+1}\beta \left(\tau \right)d\tau \right)$, *I* being the number of infectious animals and t_i_ and t_i+1_ corresponding to the first and last contact days of contact group CDi. The number of new infections during this interval follows a binomial distribution with parameters S = 8 (number of susceptible individuals at each contact trial) and pi = 1-qi. The log likelihood of this binomial distribution is given by the expression $\sum_{i=1}^{7}\left(\log\left(\begin{array}{c} S\\ Ci \end{array}\right)+Ci\log\left(pi\right)+\left(S-Ci\right)\log\left(qi\right)\right)$, where *Ci* is the number of cases in each contact trial. The time-dependent transmission parameter β(τ) was estimated by adapting the algorithm used previously in a study on Porcine Circovirus type 2 transmission [23] to our PRRSV infection data in the different contact groups. The infectious potential β(τ) was estimated by maximizing the log-likelihood function. The integrals were computed using the *quad* (quadratic approximations of integrals) function in Matlab software [24] and the log likelihood was maximized using the “quasi-Newton line search” algorithm displayed by the *fminunc* function (unconstrained minimization of a multivariable function) in the Matlab software. Confidence intervals for the parameter estimates of the β(τ) function were derived from the Hessian matrix of the parameters provided in Matlab’s *fminunc* function.

The mean disease generation time, which, by definition, is the mean time for a newly infected individual to infect a susceptible one [25], can also be computed, $Tg=\int_{0}^{\infty }\left(\tau \beta \left(\tau \right)/\int_{0}^{\infty }\beta \left(s\right)ds\right)d\tau$[26].

### Statistical analyses

Statistical analyses were performed using SAS software 9.1 [27]. The rectal temperatures, viral genome load in blood, viral shedding and total and neutralizing antibodies of pigs inoculated IC, IWC and control pigs were compared by considering the effect of “time” as a repeated effect (generalized linear model using Generalized Estimating Equation, GEE, Proc GENMOD, SAS 9.1). Multiple comparisons were performed using the Tukey’s test. The significance level chosen was *p* ≤ 0.05.

Correlations between the infectiousness estimated using the model and biological parameters (viremia, viral shedding, antibodies) were calculated, taking into account the existence of temporal correlations between the measures and the fact that the biological parameter and infectious potential were evaluated on the same individual for a given time (time-repeated, paired measurements, Proc MIXED, SAS 9.1) [28].

## Results

### Clinical findings

**Figure 2:**
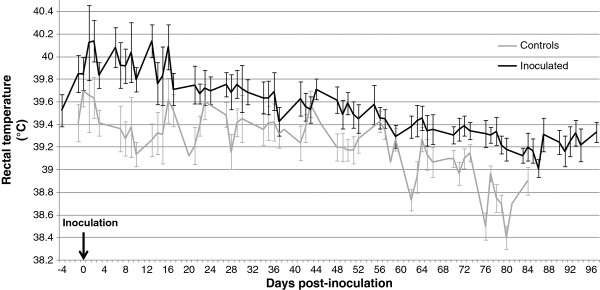
**Evolution of the averagerectal temperature of the16 inoculated (IC +** **IWC) and negative controlpigs with time post-inoculation.** Mean with standard deviation of the daily rectal temperature. Black line: infected piglets; grey line: negative control piglets.

Inoculated pigs showed clinical signs of PRRS (coughing and sneezing) 2 days after inoculation. Four piglets showed, at least for one day, high hyperthermia ≥ 41°C the day after or two days after inoculation. Because of the physiological development of inoculated piglets and negative control piglets, the average rectal temperatures decreased naturally with age (Figure 2). Despite this and taking into account repeated measurements with time, the average rectal temperatures of inoculated pigs were significantly higher than those of negative control pigs (*P* < 0.05) on the overall period but mainly due to important differences in the early times post-inoculation. No significant differences were observed between IC and IWC groups (data not shown).

### Genome load in blood and shed measured by rRT-PCR

No other tested swine virus or *Mycoplasma* was detected with the developed rRT-PCR, indicating its good specificity. The efficiency of the rRT-PCR was tested for the two target genes: PRRSV ORF-7 and beta-actin. Similar efficiency, close to 100% for each gene, was obtained (efficiency of 107% for ORF-7 and 104% for beta-actin). This simplifies the equation used in the ΔΔCt method since R can be fixed at 1. The equation then becomes R = 2^-ΔΔCt^[29]. The repeatability and reproducibility of this amplification by RT-PCR were verified.

**Figure 3:**
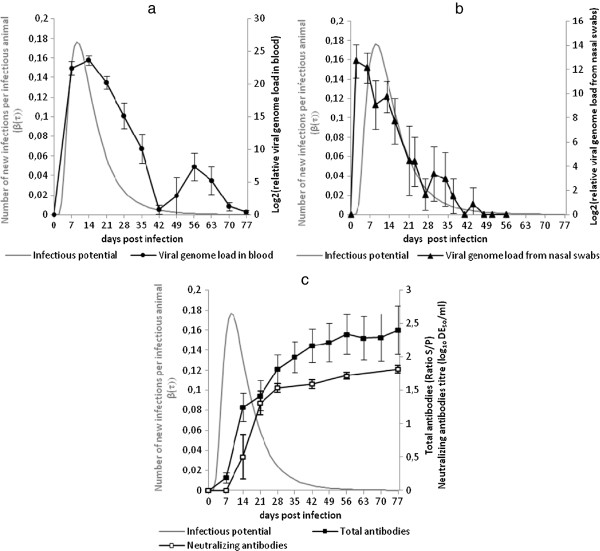
**Evolution of the estimatedinfectious potential in relationwith biological parameters inthe 16 inoculated pigs(IC + IWC).** Comparison with the genome load in blood (**a**), genome load from nasal swabs (**b**), total and neutralizing antibody titers (**c**). The grey curve represents the estimated infectious potential (β(τ)) defined as the number of new infections produced by an infectious animal per unit of time.

The 7 negative control pigs remained negative throughout the trial. No significant difference was observed between the animals in the IC and IWC groups. Viral RNA was detected in the blood of all inoculated animals from 7 dpi reaching a peak at 14 dpi and then decreasing to become negligible at 77 dpi (Figure 3a). A further apparent increase was observed at 56 dpi, however the estimated genome loads were not statistically different at 42, 49 and 56 dpi. Only the genome load at 56 dpi was different from the very low genome load measured on later samples. The inoculated animals shed viral genome as early as 2 dpi. At that time, the relative genome load was maximal and then decreased regularly to become negligible at 49 dpi (Figure 3b).

### Serology

The negative control pigs remained negative throughout the trial. No significant difference between the serological results of the IC and IWC groups was observed. Total antibodies were detected at 7 dpi in some inoculated pigs and all animals seroconverted the following week. The relative amount of total antibodies increased until 56 dpi when it stabilized at an average ratio S/P = 2.4 until the end of the experiment (Figure 3c). Neutralizing antibodies (NAbs) were detected from 14 dpi in some inoculated pigs (6/16) and at 21 dpi in all the animals except one that did not show any NAbs before 28 dpi. The amount of neutralizing antibodies then increased rapidly until 28 dpi and more steadily from that date until 77 dpi when the titer attained 1.8 log10 DCP_50_/mL (Figure 3c).

### Observed infections in contact groups

**Figure 4:**
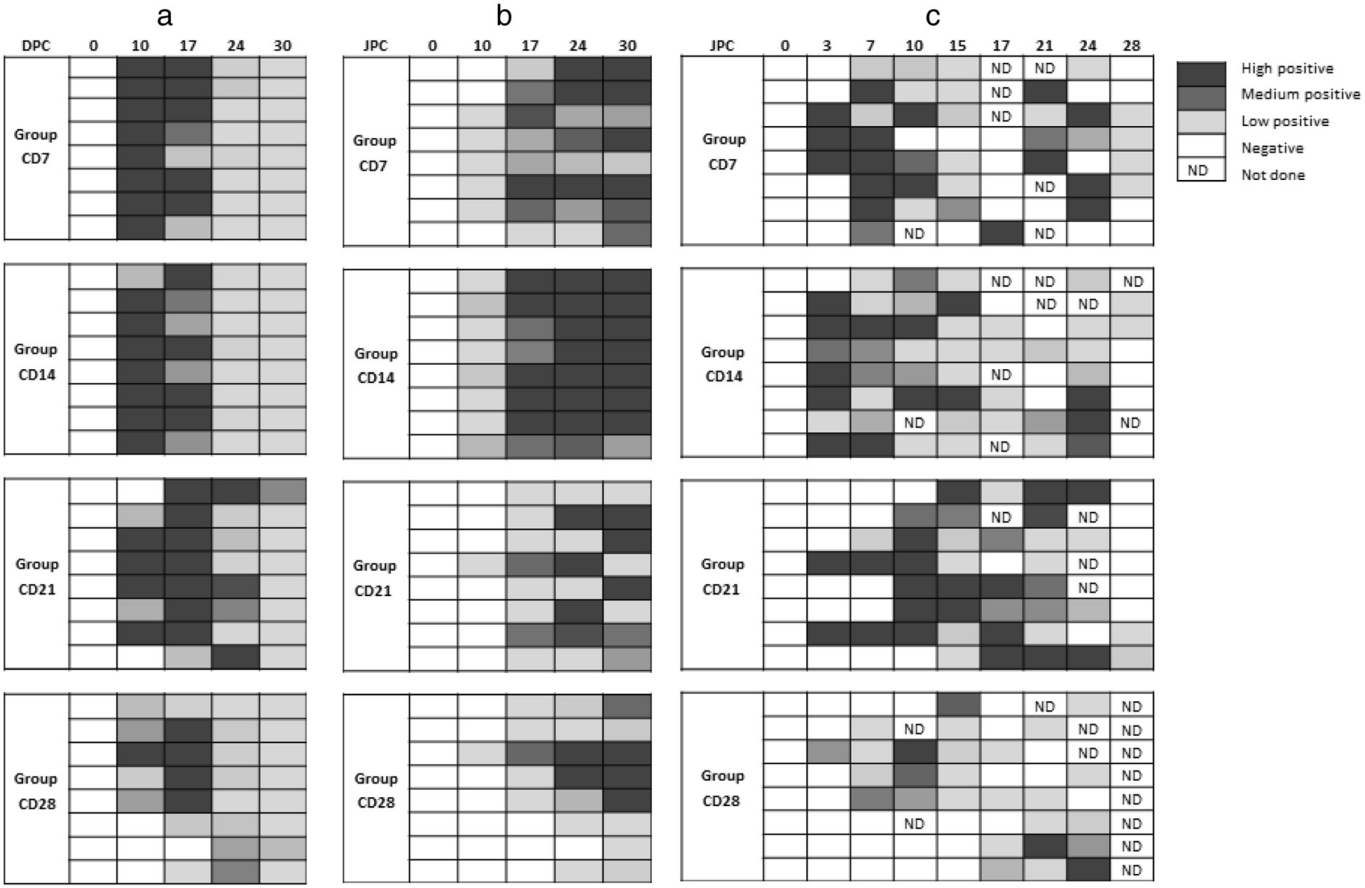
**Kinetics of observed infectionsin individual contact pigsfor groups CD7, CD14,CD21 and CD28.** (**a**) Blood viral genome load (**b**) Total antibodies (**c**) Viral genome load in nasal swabs. The qualifications as low, medium or high positive are based on the values of the 33^rd^ and 66^th^ percentiles of the distribution of relative genome load and the S/P ratio for total antibodies.

Three types of infections in contact groups were defined, based on the results of the viral genome load in blood and nasal swabs, serology and gross pathology observed during necropsies. Primary infections were defined as those occurring during the two days contact with inoculated pigs, whereas secondary and tertiary infections took place once the contact groups had returned to their own room. Viral genome shedding was the most discriminatory biological parameter able to differentiate the types of infection (Figure 4). It was assumed that pigs that did not shed virus as soon as day 3 post-contact had not been infected by inoculated pigs and that later shedding resulted from secondary infections because of the following:

(i) all the inoculated pigs shed the virus from the second day post-inoculation,

(ii) the contact rate between inoculated and contact pigs could reasonably be assumed to be uniform.

Hence, primary infections were defined as animals shedding the virus from their third day post-contact. Under this assumption, the numbers of primary infected pigs were 3, 7, 2 and 1 in groups CD7, CD14, CD21 and CD28 respectively. Pigs from groups CD42, CD56 and CD63 showed no sign of infection until slaughter (28 days after contact) either at necropsy, or from analysis of weekly blood samples or nasal swabs, which indicated that the inoculated pigs were no longer infectious at least from 42 dpi.

### Transmission parameter estimation

Assumptions were made as to the shape of the most probable infectious curve according to the number of pigs infected within each contact group. The curve was deemed unimodal, with the mode between 7 and 14 dpi. Moreover, the function was deemed to be left skewed, lower bounded, and upper bounded since only one of the eight susceptible pigs in group CD28 had been infected (primary), and none was in group CD42. According to these assumptions, three function shapes were tested,

(i) a gamma-like function defined by: $\beta \left(\tau \right)=\{\begin{array}{ll} R_{0}f_{\gamma }\left(\tau -Lat,k,\theta \right) & \text{if}\;\tau \;\geq \;Lat\\ 0 & \text{if}\;\tau <Lat \end{array}$

(ii) a lognormal-like function: $\beta \left(\tau \right)=\{\begin{array}{ll} R_{0}f_{\mathrm{Ln}}\left(\tau -Lat,k,\theta \right) & \text{if}\;\tau \;\geq \;Lat\\ 0 & \text{if}\;\tau <Lat \end{array}$

(iii) a Weibull-like function: $\beta \left(\tau \right)=\{\begin{array}{ll} R_{0}f_{\omega }\left(\tau -Lat,k,\theta \right) & \text{if}\;\tau \;\geq \;Lat\\ 0 & \text{if}\;\tau <Lat \end{array}$

where fγ, f_Ln and_ f_ω_ are the gamma, lognormal and Weibull probability density functions, respectively, with parameters k and θ, and with Lat, the duration of latency. k and θ are, respectively, the shape and scale parameters of the gamma and Weibull distributions and the mean and variance of the lognormal distribution. Thus the characterization of β(τ) requires the estimation of three parameters, k, θ and *R*_0_.

Three latency durations (0, 1 and 2 days) were tested. Table 1 summarizes the estimated parameters for latency duration between 0 and 2 dpi. A sensitivity analysis was carried out to determine the most accurate shape for β(τ). The lognormal-like function gave the best quality-of-fit when compared with the observed number of primary infections in each contact group. Although the quality-of-fit test (chi-2 and coefficient of determination) gave slightly better results with a 2-day latency duration, viral shedding was found to be maximal as early as the second day post-infection. Hence, animals were likely to already shed the virus in sufficient amounts to infect a contact pig from 2 dpi, suggesting that the estimations corresponding to the 1-day latency duration could be retained. The obtained function β(τ) (lognormal shape) showed a mode at 9 dpi and was found to be < 0.001 beyond 48 dpi (Figure 3). The basic reproduction ratio (*R*_0_) for PRRSV infection was estimated by integrating β(τ) over the entire infectious period: $R_{0}=\int_{0}^{\infty }\beta \left(\tau \right)d\tau =2.6\left[1.8,3.3\right]$. The mean disease generation time (mean time for a newly infected animal to infect a susceptible one) could also be computed, $Tg=\int_{0}^{\infty }\left(\tau \beta \left(\tau \right)/\int_{0}^{\infty }\beta \left(s\right)ds\right)\;d\tau =14.5\;\text{days}\;\left[14.4,14.6\right]$.

**Table 1:** Sensitivity analysis on the parametric shape of the function representing the time-dependent transmission rate

**Function shape**	**Latent period (days)**	**Parameters (IC 95%)**			**Mean generation time Tg(IC)**	**Goodness of fit testson observed data**	
		**K**	**θ**	***R***_**0**_		**χ**^**2**^***P*-value**	**Coefficient of determination (R**^**2**^**)**
**Gamma**	0	10.0 (−3.4, 23.4)	1.5 (1.1, 1.8)	2.4 (1.8, 3.0)	14.7 (ND, 32.3)	0.870	0.950
	1	8.7 (−0.9, 18.4)	1.6 (1.2, 1.9)	2.4 (1.8, 3.0)	14.7 (ND, 33.1)	0.878	0.953
	2	7.6 (0.8, 14.3)	1.7 (1.3, 2.1)	2.4 (1.8, 3.1)	14.7 (3.0, 30.7)	0.888	0.956
**Lognormal**	0	2.63 (2.63, 2.64)	0.31 (0.31, 0.32)	2.5 (1.8, 3.2)	14.6 (14.5, 14.7)	0.975	0.968
	1	2.5 (2.5, 2.6)	0.34 (0.33, 0.34)	2.6 (1.8, 3.3)	14.5 (14.4, 14.6)	0.979	0.971
	2	2.46 (2.45, 2.47)	0.36 (0.36, 0.37)	2.6 (1.8, 3.4)	14.5 (14.4, 14.6)	0.983	0.974
**Weibull**	0	16.4 (14.2, 18.6)	2.9 (2.5, 3.3)	2.2 (1.8, 2.6)	14.6 (12.6, 16.7)	0.629	0.874
	1	15.4 (13.3, 17.5)	2.8 (2.4, 3.1)	2.2 (1.8, 2.6)	14.7 (12.8, 16.7)	0.637	0.880
	2	14.4 (12.4, 16.4)	2.6 (2.4, 2.9)	2.2 (1.8, 2.6)	14.8 (13.0, 16.6)	0.647	0.887

Estimates and 95% confidence intervals (CIs) of the parameters of the infectious potential function distribution, estimated basic reproduction ratio *R*_0_, mean generation time Tg and goodness of fit tests on observed data according to the assumed duration of the latent period.

(β(τ)), parametric function of the infectious potential; k, θ, parameters of β(t); *R*_0_, basic reproduction ratio defined as the average number of secondary infections occurring from a single infected animal during its infectious period in a totally susceptible population; Tg, mean generation time defined as the sum of the mean latent period and the mean infectious period; R^2^, coefficient of determination; ND, not defined).

The correlations between the infectiousness estimated from the model and virological and serological parameters were calculated over the entire infectious period, for the growth phase only (≤ 9 days) and for the decreasing phase of infectivity only (> 9 days). The evolution of infectiousness was mainly correlated with the time-course of viral genome load in blood (correlation coefficient = 0.82) whereas the decrease in infectiousness was related to the increase in total antibodies (correlation coefficient = −0.53).

## Discussion

This study has produced original data on PRRSV transmission and dynamics of infection which increases our understanding of PRRS epidemiology in pig populations. The biological results obtained in inoculated pigs showed that the kinetics of blood viral genome load was consistent with descriptions available in the literature [30]. After a rapid increase in genome load, the peak was reached two weeks after infection followed by a gradual decrease until 90 dpi. In our study, a further increase of viral genome load was observed at 56 dpi, which has not been previously described in the literature, to the best of our knowledge. However, as there were no statistically significant differences between the viral genome loads at 42, 49 and 56 dpi, these results mainly suggest that there was still a significant genome load at 56 dpi. This observation merits confirmation in further investigations over a similar period using more animals. However, the results from the contact groups CD56 and CD63 suggested that this supposed new increase in virus activity was not sufficient for the inoculated pigs to infect other animals. In addition, the amount of shed viral genome (nasal swabs) at that time was very low. In our study, the viral genome load shed by inoculated piglets increased very rapidly, in agreement with previous studies [31], reaching a maximum at 2 dpi and then decreasing steadily until 48 dpi when it was negligible.

Seroconversion was observed in some pigs at 7 dpi and a week later in all animals, which was consistent with previous research conducted on this virus [32-34]. The amount of antibody increased until 56 dpi and subsequently stabilized. In contrast to other studies in which a late appearance of neutralizing antibodies (NAbs) was observed [33-35], NAbs were detected as early as the second week after inoculation in some pigs and in all pigs at 21 dpi (except for one that did not have any neutralizing antibodies before 28 dpi). In another study, neutralizing antibodies were only detected from 56 dpi onwards [32]. The neutralizing antibody titer was relatively low (maximum titer in log_10_ of 1.8 at 77 dpi) in agreement with previous findings [9]. No significant difference was observed between the IC and IWC groups for the virological and serological parameters studied. Hence, the stress due to the successive contact trials with different animals did not affect the serological response of inoculated pigs or promote the replication of virus and viral shedding.

The principle of this study was based on 7 successive contact trials between groups of susceptible and inoculated pigs at different times post-inoculation. This approach differs from other experiments on PRRS virus transmission which all used the final size algorithm to estimate transmission parameters [36,37]. Most of them gave disappointing results, particularly when the duration of the experiment was too short compared to the actual duration of the infectious process or when all the contact animals were found infected at the end of the trial. In our study, the number of primary infected animals increased up to 87.5% (susceptible animals infected at 14 dpi) then decreased and no contact animal was found infected at 42 dpi. Since a primary infection took place at 28 dpi, running a contact trial at 35 dpi would have probably been informative. The results would certainly have helped to determine more precisely the date when pigs no longer shed infectious particles in a sufficient quantity to infect susceptible individuals, especially as the results of PCR on nasal swabs did not indicate a total termination of viral genome shedding before 48 dpi. It cannot be excluded that a primary infection could have occurred in a contact at 35 dpi. Various data (results from serology, viral genome shedding, viral genome load in blood and gross lesions observed at necropsy) were analyzed to differentiate primary from secondary and tertiary infections. However, the most informative results were the analyses of nasal swabs collected every 3 days which provided the most precise date from which the pigs started to shed virus. Indeed, the first blood sample was taken only 10 days after the first day of contact and failed to differentiate primary from secondary infections in groups CD7 and CD14, based on blood genome load or serological results, since all the animals already tested positive at that sampling time. Because PRRSV has a very short incubation period, it is not sure whether additional blood samples at 3 dpi and 7 dpi would have been able to discriminate between primary and secondary infections. Some authors detected the appearance of genome load in blood at 3 or 7 dpi but only in some pigs [38,39] and seroconversion usually occurs between 5 and 14 dpi [32-34]. In addition, taking blood samples very frequently increases the risk of hematoma at the collection site, which is not ethically acceptable. Since all inoculated animals shed viral genome at 2 dpi and the contact rate between inoculated and contact pigs could reasonably be assumed to be uniform, it was assumed that primary infections corresponded only to animals shedding the virus from their third day post-contact. The only method that would have allowed us to be absolutely certain of the number of primary infections in the groups would have involved separating individual animals from each contact group after the contact. This was not feasible given the number of animals used for the study. Each room contained two pens of four pigs with a polyethylene separation between the pens. However, the group CD28 had only one primary infection (in one pen) and pigs that were not located in that pen still ended up being infected. This suggests the possibility of cross-contamination between pens that were 40 cm apart and did not allow the pigs to have direct contact.

By adapting the algorithm used by Andraud et al. [23] to the numbers of primary infections in each contact group, we were able to estimate the infectiousness of PRRSV infected animals with time since infection. We obtained a distribution of infectiousness with an average duration of infectiousness of 14.8 days, peak infectivity at 9 dpi and a negligible probability of transmission beyond 48 dpi. These results suggest that the infectious period was shorter than that obtained by other authors who reported up to 56 dpi [40] or 62 dpi [41]. The major differences compared to the above studies are due to detailed characterization of the evolution of this infectious potential with time, which definitely cannot be considered constant. Even though a viral genome load could be detected beyond 48 dpi, the results from contact trials (which are the direct evidence of virus transmission) showed that pigs were not able to infect others at that time. These estimates are specific to the strain we used (genotype I, subtype 1), and significant differences might be expected with strains having different pathogenic characteristics and/or a different host immune response, such as genotype II strains or even other subtypes of genotype I strains [42]. The correlations established between virological parameters and transmission characteristics could however be used to assess the transmission features of other strains according to virological and immunological data. Even if this strain is representative of our regional field situation, differences might be expected in other areas where a larger panel of strains can be involved. In addition, further work should also be carried out to assess the impact of multiple infections by different strains on transmission.

In our study, the rapid increase in infectiousness from 1 to 9 dpi was related to the increase of blood viral genome load, and to a lesser extent with the shed viral genome load which started earlier. Infectiousness was better correlated with viral genome load in the blood, over the entire period of the study, than with the shed genome load (corr = 0.82 versus 0.59 respectively). However when considering only the decreasing part of the infectiousness curve, the correlation was stronger with the viral genome shed in nasal swabs which ended simultaneously to transmission termination. Several assumptions can be made about the observed delay between viral genome shedding and significant increase of the infectious potential. First, it is possible that infected animals shed defective interfering viral particles at the early times (spontaneous generation of mutant viruses in which an essential part of the viral genome had been removed, making it non-functional) [43]. Another hypothesis is that the infectiousness of the shed viral particles differs over time with particles shed at 9 dpi being more infectious than those shed at 2 dpi. It is also possible that at the peak of infectiousness, the virus was shed by other routes in addition to nasal secretions such as feces, saliva or urine [44]. The decrease in infectiousness at 9 dpi also corresponds to the time when the amount of total antibody and neutralizing antibody increased. The decrease in infectiousness was slightly less correlated with the amount of neutralizing antibodies than with the amount of total antibodies (corr = −0.47 compared with −0.53 respectively). However, this might be due to the fewer dates when neutralizing antibodies could be titrated. The role of neutralizing antibodies in protection against PRRSV is controversial. Even if a link between the development of neutralizing antibodies and viral clearance has been described [45], most studies have shown that the protection provided by neutralizing antibodies after experimental infection is very low and irregular with great individual variability [32,33]. The results from our study suggest that neutralizing antibodies could help to control infectiousness even though a significant viral genome load persisted in the blood in their presence. Further work has to be done to assess the contribution of the cell-mediated immune response on infectiousness control.

The estimated *R*_0_ in this study is a theoretical parameter, indicating how the virus might spread in a fully susceptible and large population. Given the structure of pig farms, the results now need to be applied to a model describing population structure and dynamics (variability between individuals, contact structures, culling and replacement of the animals). The estimated basic reproduction ratio of PRRSV was 2.6, which is quite low compared to the *R*_0_ estimates available for other viral infections in pigs (100 for Classical Swine Fever virus [46], 5.9 for the PCV2 virus [23], 8.8 for hepatitis E [47]). The relatively small *R*_0_ value for PRRSV together with the relatively long duration of infectiousness can be explained by the huge variations in transmission rate with time-since-infection, as shown in the present study. However, the value obtained is significantly higher than 1, which suggests that the virus is gradually but efficiently spread and maintained in a structured population.

## Competing interests

The authors declare that they have no competing interests.

## Authors’ contributions

CC developed and validated the rRT-PCR, performed the sample analysis, analysed the data and drafted the manuscript, SM developed the rRT-PCR and performed virological and serological analyses, AK and RC supervised the experiment, CB participated in the design, MFLP supervised the laboratory work and participated in the coordination of the study, NR conceived and coordinated the study, participated in the statistical analyses and writing the manuscript. All the authors read and approved the final manuscript.
